# Effects of different floor materials on the welfare and behaviour of slow- and fast-growing broilers

**DOI:** 10.5194/aab-62-335-2019

**Published:** 2019-06-14

**Authors:** Enver Çavuşoğlu, Metin Petek

**Affiliations:** Department of Animal Science, Faculty of Veterinary Medicine, Bursa Uludag University, Bursa, Turkey

## Abstract

Litter quality and fast growth rate are the two main issues in broiler welfare. This study aimed to evaluate the effects of genotype and floor material on broiler welfare and behaviour. In the study, slow-growing (Hubbard JA57) and fast-growing (Ross 308) broilers on a slatted floor and deep litter were used; there were four main groups (2 genotype × 2 housing) and each treatment group consisted of 5 replicates. Each replicate group consisted of 10 male chicks, and 200 birds were used in total. The experiment lasted for 8 weeks. The welfare parameters were recorded in weeks 6, 7, and 8, whereas behaviour data were collected in week 8 only. Results showed that welfare parameters of broiler chickens were affected by genotype and floor type. Slow-growing broilers had better welfare parameters than fast-growing broilers. The slatted floor had a positive effect on main welfare parameters of the birds. Slower-growing broilers had a longer distance in the avoidance distance test. Tonic immobility reaction was longer in slow-growing broilers compared to fast-growing broiler. On the other hand, floor type did not affect behaviour parameters. As a conclusion, slow-growing broilers had better welfare parameters than fast-growing broilers and slat flooring could be beneficial to improve broiler welfare, but further behavioural investigations are needed such as dust bathing and walking behaviour.

## 1 Introduction

Rapidly growing chickens in broiler meat production have been intensively selected for 40–50 years, and performance of broiler chickens has improved rapidly by the developments of feeding, environmental conditions, and health improvements (Fanatico et al., 2007). Nowadays, a broiler chicken can reach up to 2.5 kg live body weight in 5 or 6 weeks of age with about 1.7 to 1.8 feed conversion ratio at the end of the growing period (Awad et al., 2009; Goliomytis et al., 2014). This fast growth rate has led to some undesirable consequences, which are one of the main concerns in animal welfare. Fast growth rate, metabolic diseases associated with fast growth rate, lower locomotor activity, high stocking density, and bad management of air and litter quality have become main welfare topics of broiler meat production in the last 20 years (RSPCA, 2017; Bessei, 2006). Fast-growing broilers show higher rates of heart attack and hypoxia (Julian, 2005; Olkowski et al., 1998; Reeves et al., 1991) and are more prone to behavioural disorders and immune system impairments (Rauw et al., 1998). These are caused by the rapid growth rate of muscle tissues of broilers which was achieved by breeding programmes and has been criticized for its negative impact on animal welfare. Therefore, either slower-growing broilers or a different feeding regime to control the growth rate has been advised in commercial broiler production (Dawkins and Layton, 2012).

In modern broiler meat production, chickens are usually housed in deep-litter barns (Berg, 2002; Bergmann et al., 2017). There are many kinds of bedding material such as riverbed sand, coconut husk, rice hulls, guinea grass, newspaper combined with wood shavings, and corncob. Litter and air quality are very important for broiler welfare since broilers spend all of their lives on litter material and their abdomens, legs, and feet are in contact with the litter. Fast-growing broilers spend most of their time sitting, especially after 3 weeks of age. Moreover, the quality of litter affects the level of air dust, litter moisture, and ammonia. High levels of ammonia in litter causes inflammation of eyes and larynges of the birds and increases mortality rate (Shepherd and Fairchild, 2010). Feed intake, body weight, and carcass weight might be reduced when the feet of the bird are in contact with manure and the litter (Chuppava et al., 2018). Wet litter is the main cause of contact dermatitis in different body regions in broiler chicks (Dunlop et al., 2016; Mayne et al., 2007; Shepherd and Fairchild, 2010). The occurrence of foot-pad dermatitis can have significant welfare and financial implications (De Jong et al., 2014).

As a result of poor animal health and welfare-related problems in deep-litter production systems, which occur when the litter management is not good enough, alternative floor systems come into question in commercial broiler meat production (Petek et al., 2015; Petek and Orman, 2013). Although cages and slatted floor housing for broiler meat production have been available for many years, they have not become common because broiler chickens are prone to leg deformities and breast blisters, which adversely affects broiler meat quality (Zhao et al., 2009). As a result of current technological improvements, cage systems have recently become popular in many countries such as Russia and Turkey (Özhan and Simsek, 2014). However, limited space and inappropriate conditions for natural broiler behaviour in cage systems have been criticized for the poor wellbeing of poultry, e.g. in egg production. It was reported that using a fully slatted floor not only led to higher body weight but also reduced the foot-pad injury rate (Chuppava et al., 2018). It has been thought that slatted floors would become more popular since they have no litter cost and they minimize the negative effects of improper litter management (Shields and Greger, 2013; Slepukhin et al., 2000; Petek et al., 2015).

Although numerous studies have been conducted to study the effect of different litter and floor materials on the welfare of broilers, slat flooring has rarely been evaluated (Chuppava et al., 2018; Kaukonen et al., 2017; Petek et al., 2015). There is also a lack of information about the influence of slat flooring on broiler behaviour and welfare. This study aimed to investigate the effects of floor material on some welfare and behaviour parameters of fast- and slow-growing broilers under experimental conditions.

## 2 Material and methods

The study was conducted at the experimental animal farm of Bursa Uludag University, Faculty of Veterinary Medicine. Ethical permission has been given for this study from Bursa Uludag University, Ethical Committee for Animal Experiments with the tracking number of 2015-10/12.

### 2.1 Management

In this study, the effects of two flooring types (deep litter and slatted floor) and two genotypes of broilers (slow-growing Hubbard JA57 and fast-growing Ross 308) were investigated. Thus, there were four main groups (2×2) and five replicates of each main group in the study. For each replicate, 1 m${}^{2}$ space was provided and 10 male chicks were put into each replicate group. Therefore, each main group consisted of 50 chicks; in total, 100 slow-growing and 100 fast-growing 1-day-old chicks were studied. The chicks were allocated to each replicate randomly at the same time. Replicates of all main groups were distributed to every part of the barn to eliminate the environmental effect on groups.

To prevent chicks falling through holes between the slats, the floor on the slats was covered by paper during the first week. Rice hull, 7 kg m${}^{-2}$, was used as litter material in deep litter. All birds in the groups were raised under standard broiler raising conditions for 8 weeks.

During the day, daylight was used as a light source, and during the dark period, tungsten lights were used. A continuous light regime consisting of daylight and artificial light was used in the first 7 days of the experiment. From the eighth day until the end of the experiment, daylight and intermittent lighting (2 h light + 2 h darkness for a total of 16 h darkness period in each day) were applied during the night. All birds were fed with a commercial multiphase feed (starter from days 0 to 15, grower I from days 15 to 30, grower II from days 30 to 40, and finisher from days 40 to 56), which was produced by a commercial feed company in Turkey.

### 2.2 Data

#### 2.2.1 Welfare parameters

In the study, a welfare assessment was performed on the birds at 6, 7, and 8 weeks of the experiment. The birds were scored on four main welfare measures: gait score (walking ability), plumage cleanliness (breast dirtiness), foot-pad dermatitis, and hock burn at the ages of weeks 6, 7, and 8.

In live birds, walking ability (gait score) was assessed using the scoring system developed by Kestin et al. (1992).

The methodology consisted of visual observations of how birds walk on a surface. The system is divided into six levels as follows: 0 (healthy bird); 1 (the bird moves fast, but a slight walking deficiency is observed); 2 (the bird moves fast, but there is significant walking deficiency); 3 (the bird moves fast, but it presents an important deficiency); 4 (the bird moves with serious difficulty); and 5 (the bird barely moves and often uses the wings for crawling).

The external examination of food pad, hock joint, and plumage was performed for all birds at the end of the sixth week, then weekly until week 8. Hock-joint dermatitis was assessed using a five-scale score in accordance with RSPCA (2017) to levels 0: no discolouration or lesions present on hocks; 0.5: less than 25 % of the hock is covered with a lesion; 1: between 25 % and 50 % of the hock is covered with a lesion; 1.5: between 50 % and 75 % of the hock is covered with a lesion; 2: more than 75 % of the hock is covered with a lesion.

Foot-pad lesions were scored according to five levels: a score of 0 indicated no lesion, 1 indicated a very small or superficial lesion, 2 indicated a mild lesion (minor superficial lesion), 3 indicated a medium-severity lesion (moderate hyperkeratosis), and 4 indicated a severe lesion (deep and large epithelial necrosis) (Welfare Quality Consortium, 2009; Butterworth, 2013; Pagazaurtundua and Warriss, 2006).

Breast plumage dirtiness was scored visually from 1 (very clean) to 8 (very dirty) as reported by Wilkins et al. (2003).

#### 2.2.2 Behavioural parameters

To assess the behaviour of the birds, tonic immobility was measured, and an avoidance distance test was performed at the end of the experiment for each bird.

*Tonic immobility*. Before applying the test, the birds were put in a separated room to avoid disturbance by other birds. This test was induced by placing the bird on its back on a flat surface area and restrained by holding one hand on its sternum for 15 s (Jones and Faure, 1981). After removing the hold of the experimenter, a stopwatch was started while the person retreated about 1.5 to 2 m away out of sight of the bird. The duration of tonic immobility, time until the bird recovers its position to normal standing, was recorded.

*Avoidance distance test*. Each bird was taken out of the group and put down on a flat area of a separated compartment and 5 min were given for the birds to rest and the test person was in sight of the bird to get used to the test person. After that, the test person stood at a distance of 1.5 m from the bird. One hand was held in front of the body, the other hand was hanging loose at the side, and then the test person approached the bird at a speed of one step per second until it withdrew and then measured the distance from the test person's hand to the position of the bird's feet before the withdrawal. That distance was calculated as the avoidance distance test value (Graml et al., 2008). The same person applied all tests to avoid the step size difference of different persons.

### 2.3 Statistical analysis

All statistical analyses for all traits investigated were performed using SPSS^®^ computer software 13.00 (IBM SPSS, 2011). Analysis of variance was used to test the effects of, and interactions between, floor type and genotype of broiler (Snedecor and Cochran, 1989). The general form of the model used in the analyses was the following:

1 Yijk=μ+Ai+Bj+A×B+eijk,

where A represents the effects of floor type and B represents the effects of genotype; A×B represents an interaction; also i=1,2 (1= deep litter, 2= slat), j=1,2 (1= fast-growing genotype, 2= slow-growing genotype). μ is a constant and e is an error term.

## 3 Results

**Figure 1:**
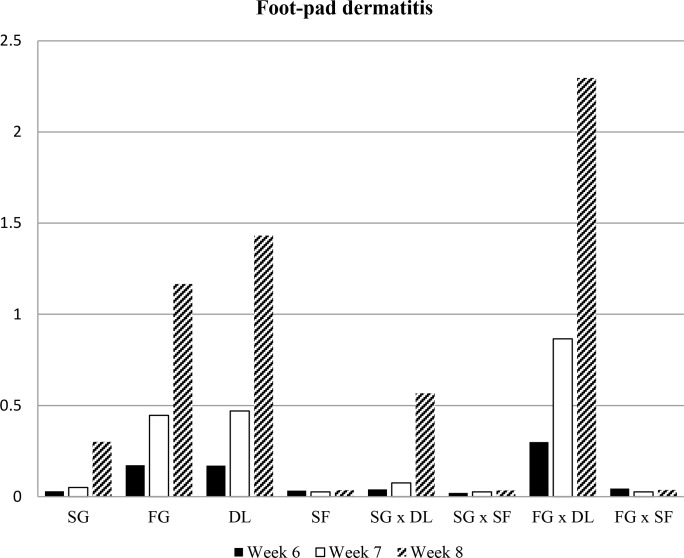
Mean value of foot-pad dermatitis scores of all groups. SG: slow growing, FG: fast growing, DL: deep litter, SF: slatted floor.

**Figure 2:**
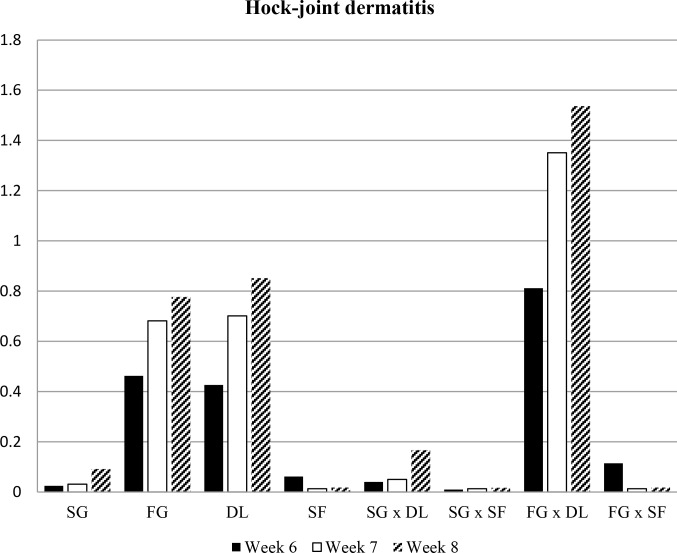
Mean values of hock-joint dermatitis scores of all groups. SG: slow growing, FG: fast growing, DL: deep litter, SF: slatted floor.

**Figure 3:**
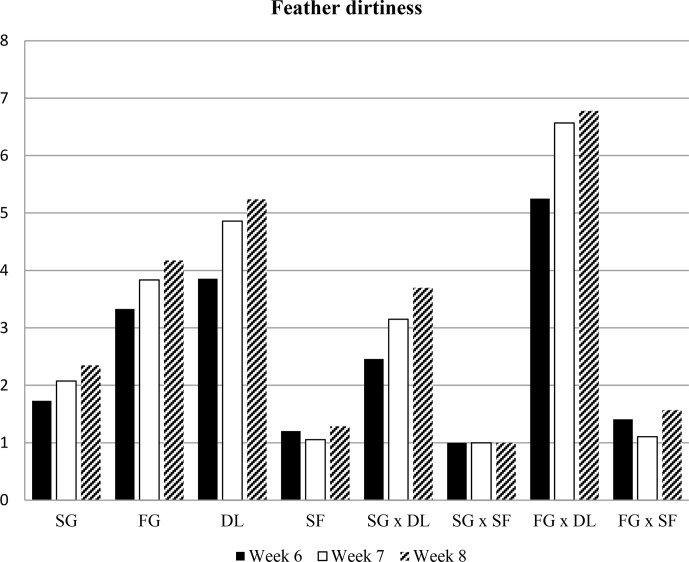
Mean values of feather dirtiness scores of all groups. SG: slow growing, FG: fast growing, DL: deep litter, SF: slatted floor.

**Figure 4:**
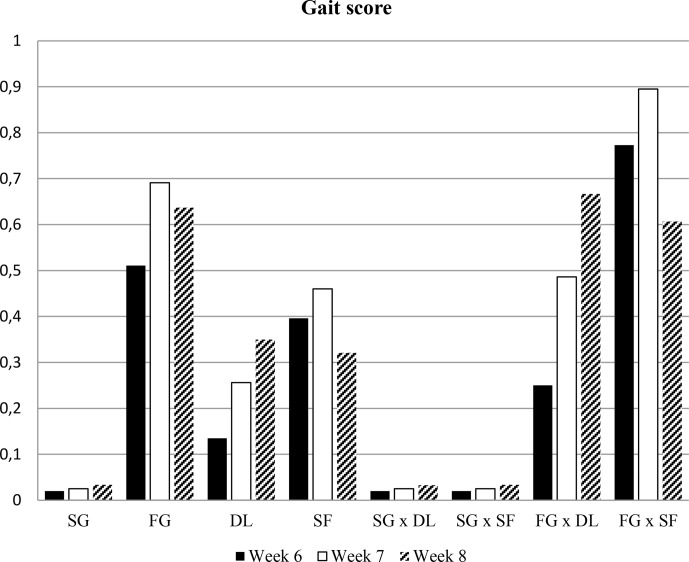
Mean values of gait scores of all groups. SG: slow growing, FG: fast growing, DL: deep litter, SF: slatted floor.

Broiler welfare was measured by assessing scores of the foot-pad dermatitis and hock-joint dermatitis, breast feather dirtiness, feather cover, and gait at the ages of 6, 7, and 8 weeks in the experiment. All welfare indicators investigated in the experiment are presented in Figs. 1, 2, 3, and 4.

### 3.1 Foot-pad dermatitis

In this study, a significant difference was observed for foot-pad dermatitis between slow- and fast-growing broilers in both flooring systems. Fast-growing broilers had a higher (worse) foot-pad dermatitis score than slow-growing broilers throughout the experiment at all ages (P<0.005, P<0.001, P<0.001). However, the highest (worst) foot-pad dermatitis scores were found in the fast-growing deep-litter group. The mean score for the birds on the slatted floor was lower than for birds raised on deep litter at all ages (P<0.007, P<0.001, P<0.001). The genotypes–floor-type interactions for foot-pad dermatitis were significant for all ages. The prevalence of scores 3 and 4 for foot-pad dermatitis existed only in fast-growing broilers in the deep-litter group (Table 1).

**Table 1:** Prevalence of foot-pad dermatitis in the experimental groups at the end of the experiment (%).

	Scores${}^{*}$				
Groups	0	1	2	3	4
Genotype					
Slow growing	76.3	15.2	8.5	0	0
Fast growing	61.8	9.1	7.3	5.5	16.4
Flooring					
Deep litter	42.1	21.1	15.8	5.3	15.8
Slatted floor	96.5	3.5	0	0	0
Genotype–flooring					
Slow growing – deep litter	56.7	26.7	16.6	0	0
Slow growing – slatted floor	96.6	3.4	0	0	0
Fast growing – deep litter	25.9	14.8	14.8	11.2	33.3
Fast growing – slatted floor	96.4	3.6	0	0	0

${}^{*}$ Welfare Quality Project, assessment protocol for poultry, 2009.

### 3.2 Hock-joint dermatitis

The prevalence of hock-joint dermatitis on the birds in the experimental groups is presented in Table 2. Broiler chickens kept on the slatted floor were characterized by significantly lower hock-joint dermatitis scores throughout the experiment. In both genotypes, hock-joint dermatitis was significantly higher when using deep-litter flooring. All animals on the slatted floor had scores <1 for hock-joint dermatitis. Hock-joint dermatitis scores for all birds of the slow-growing broilers were below 1.5 at the end of the experiment (Table 2). Fast-growing broilers housed in deep-litter flooring had significantly higher hock-joint dermatitis than the other subgroups (Fig. 2).

**Table 2:** Prevalence of hock-joint dermatitis in the groups in week 8 (%).

	Scores${}^{*}$				
Groups	0	0.5	1	1.5	2
Genotype					
Slow growing	83.1	13.6	3.4	0	0
Fast growing	49.1	10.9	7.3	1.8	30.9
Flooring					
Deep litter	38.6	19.3	10.5	1.8	29.8
Slatted floor	94.7	5.3	0	0	0
Genotype–flooring					
Slow growing – deep litter	70	23.3	6.7	0	0
Slow growing – slatted floor	96.6	3.4	0	0	0
Fast growing – deep litter	3.7	14.8	14.8	3.7	63
Fast growing – slatted floor	92.9	7.1	0	0	0

${}^{*}$ RSPCA broiler welfare assessment protocol, 2017.

**Table 3:** Breast feather dirtiness score prevalence in week 8 (%).

	Scores${}^{*}$							
Groups	1	2	3	4	5	6	7	8
Genotype								
Slow growing	49.2	6.8	16.9	13.6	11.9	1.4	0	0
Fast growing	25.5	21.8	3.6	0	1.8	20	14.5	12.7
Flooring								
Deep litter	0	7	17.5	14	14	21.2	14	12.3
Slatted floor	75.4	21.1	3.5	0	0	0	0	0
Genotype–flooring								
Slow growing – deep litter	0	13.3	33.3	26.8	23.3	3.3	0	0
Slow growing – slatted floor	89.7	10.3	0	0	0	0	0	0
Fast growing – deep litter	0	0	0	0	3.7	40.7	29.7	25.9
Fast growing – slatted floor	50	42.9	7.1	0	0	0	0	0

${}^{*}$ Wilkins et al. (2003).

### 3.3 Feather dirtiness scores

The prevalence of breast feather dirtiness on the birds in the experimental groups is shown in Table 3. Slow-growing broiler breast feathers were cleaner compared to feathers of fast-growing broilers through the experiment. As expected, the birds raised on the slatted floor had much cleaner feathers than those raised on deep litter. The interaction between genotype and floor type was found to be significant in weeks 6, 7, and 8. While only 12.3 % of slow-growing broilers had scores of 5 and higher, 49 % of fast-growing birds had scores of 5 and higher. No birds raised on slatted floors had scores 5 or greater, but 61.5 % of the birds in the deep litter had scores 5 and higher. The fast-growing broilers raised in the deep litter had the dirtiest feathers among all groups.

### 3.4 Gait scores

In this study, all the slow-growing broilers had better gait scores compared to the fast-growing broilers. Even though the birds in the deep litter had a better walking ability in week 6, there were no statistical differences between the two floor types in weeks 7 and 8. Genotype and floor type interaction for gait were only significant in week 6 of the growth period. The birds in the fast-growing–slatted-floor group had the poorest gait score in week 6 (Fig. 4). The prevalence of gait scores in the experiment is shown in Table 4. All the birds in slow-growing broilers had only gait score 0 and 1. However, 18.1 % of fast-growing broilers had gait score 2 and greater. When subgroups were compared, 14.8 % of the fast-growing–deep-litter group had score 2 or higher.

**Table 4:** Prevalence of gait scores in the groups in week 8 (%).

	Scores${}^{*}$					
Groups	0	1	2	3	4	5
Genotype						
Slow growing	96.6	3.4	0	0	0	0
Fast growing	70.9	10.9	10.9	1.8	1.8	3.6
Flooring						
Deep litter	82.5	10.5	3.5	0	1.8	1.8
Slatted floor	86	3.5	7	1.8	0	1.8
Genotype–flooring						
Slow growing – deep litter	96.7	3.3	0	0	0	0
Slow growing – slatted floor	96.6	3.4	0	0	0	0
Fast growing – deep litter	66.7	18.5	7.4	0	3.7	3.7
Fast growing – slatted floor	75	3.6	14.2	3.6	0	3.6

${}^{*}$ Kestin et al. (1992).

### 3.5 Behaviour

Results on the avoidance distance test and tonic immobility are shown in Table 5. While slow-growing broilers had 22.16 cm avoidance distance, fast-growing broilers had only 3.4 cm avoidance distance. Additionally, slow-growing broilers had a longer tonic immobility reaction period (122.39 s) compared to fast-growing broilers (36.31 s). There was no significant difference between the two flooring types in both avoidance distance test and tonic immobility test results.

**Table 5:** Avoidance distance test and tonic immobility test scores in the groups (mean ± SEM, standard error of the mean).

Groups	Avoidance distance	Tonic
	test (cm)	immobility (s)
Genotype		
Slow growing	22.16±2.37	122.39±20.60
Fast growing	3.40±2.64	36.31±22.96
Flooring		
Deep litter	10.88±2.57	75.94±22.36
Slatted floor	14.68±2.44	82.75±21.24
Genotype–flooring		
Slow growing – deep litter	19.40±3.59	132.05±31.22
Slow growing – slatted floor	24.92±3.09	112.74±26.87
Fast growing – deep litter	2.36±3.68	19.84±32.03
Fast growing – slatted floor	4.44±3.78	52.77±32.91
ANOVA		
	P value	P value
Genotype	0.001	0.007
Flooring	0.287	0.826
Genotype–flooring	0.628	0.400
	Degree of freedom	Degree of freedom
Genotype	1	1
Flooring	1	1
Genotype–flooring	1	1
	F value	F value
Genotype	21.948	7.786
Flooring	1.148	0.049
Genotype–flooring	0.236	0.717

## 4 Discussion

Foot-pad dermatitis and hock-joint dermatitis, which are a form of contact dermatitis, are commonly observed in fast-growing chickens in broiler meat production. Both negatively affect the welfare of birds and performance parameters in poultry meat production (Grandin, 2017). Foot-pad dermatitis is not only important for broiler welfare but also for production economics (Bokkers and de Boer, 2009) since foot-pad dermatitis causes pain and pain impedes animals to reach the feed. Moreover, foot-pad dermatitis also causes hock-joint dermatitis and breast blisters, and this reduces the profitability (De Jong et al., 2014).

Several factors affect the foot-pad condition (Shepherd and Fairchild, 2010). Unsuitable or irritating litter and litter materials are considered the most important risk factor for contact dermatitis (Bessei, 2006; Haslam et al., 2007), and it can be painful and affect walking ability (Taira et al., 2014; Zikic et al., 2017). The presence and severity of foot-pad and hock skin lesions in broilers are considered to reflect housing conditions. De Jong et al. (2018) reported that on-farm hatched flocks had less foot-pad dermatitis, which indicated better welfare. In addition to the damaging effect of litter material on foot-pad skin (Bassler et al., 2013; De Jong et al., 2014), damp litter also reduces the dust bathing of broilers (Moesta et al., 2008). Moreover, wet litter results in dirty plumage (Martland, 1985) and decreases broiler growth and feed efficacy (De Jong et al., 2014). Nutrition is an important factor affecting water intake, excreta moisture, and litter quality and, in this way, the occurrence and intensity of foot-pad dermatitis in birds (Cengiz et al., 2013). Meluzzi et al. (2008) found that the incidence of foot-pad dermatitis is higher in flocks reared in winter than those reared in the summer because the ventilation rate, reduced to maintain the temperature, is not enough to remove the excess of moisture from the air and litter. It is observed that a high incidence of foot-pad lesions in birds kept at a stocking density of 35 kg m${}^{-2}$ is associated with a high litter nitrogen content, which causes a lower litter pH (Meluzzi et al., 2004).

In this study, the majority of birds raised on slatted floor, and the majority of slow-growing broilers showed no foot-pad dermatitis or hock-joint dermatitis (score 0). In contrary, there was a lot of foot-pad dermatitis in fast-growing broilers raised in deep litter (Table 1). In broiler production, breed and cross-breeds can differ in their susceptibility to foot-pad dermatitis. In particular, slow-growing breeds have been shown to be less susceptible to foot-pad dermatitis compared to fast-growing strains and cross-breeds (Bilgili et al., 2006; Çavuşoglu et al., 2018; Kestin et al., 1992; Kjaer et al., 2006; Sarica et al., 2014; Shepherd and Fairchild, 2010). Whilst it has been shown that male birds are more susceptible than females to foot-pad dermatitis (Bilgili et al., 2006; Nagaraj et al., 2007), there is also evidence to suggest the opposite (Kjaer et al., 2006), and it may be that body weight is a more important risk factor than sex (Dawkins et al., 2017). It was reported that the prevalence of hock burns was lower in lighter weight birds, mild hock burns and mild foot-pad dermatitis were more common in medium weight birds, and severe hock burns were more frequent in heavier birds (Dawkins et al., 2017). There is a significant interaction between genotype and floor type for foot-pad dermatitis. The negative effect of deep litter for this parameter was only linked to fast-growing broilers, as suggested by another research report (Sarica et al., 2014). Our experiment suggests that deep litter would be more beneficial for slow-growing broilers.

In the current study, the hock-joint dermatitis results were similar to the prevalence of foot-pad dermatitis across the groups (Table 2). A total of 50.90 % of fast-growing broilers had hock lesion scores 0.5 and higher. Additionally, 7.30 % of them had mild hock burns (score 1) and 30.9 % of the birds had severe hock burns (score 2). These values are higher than those found for moderate or severe hock lesions by Haslam et al. (2007) and Hepworth et al. (2011). The significant genotype–floor-type interactions for hock lesions revealed that only the birds in the fast-growing–deep-litter group showed a greater incidence of severe hock-joint dermatitis (score 2; 63 %). According to Kjaer et al. (2006) and Sørensen et al. (2000), the high prevalence of hock burns in heavier birds may be related to the fact that they spend more time lying on their joints as compared to lighter birds. In another study, no differences in hock and foot-pad lesions and lameness on different floor types were found (Li et al., 2017). The higher incidence of hock lesions on deep litter might be caused because this material is more abrasive than plastic floors that have a smoother surface (Haslam et al., 2006).

Breast feather dirtiness is one of the basic indicators of environmental conditions in a broiler house (Saraiva et al., 2016). It is correlated with contact dermatitis and lameness within the individual in broiler meat production. As expected, in this study, the birds raised on slatted floors had much cleaner feathers than those raised in deep litter (Wilkins et al., 2003). The fast-growing birds raised in the deep litter had much dirtier feathers than slow-growing birds raised in deep litter. The interaction between genotype and floor type for breast dirtiness was significant in weeks 6, 7, and 8. This was because fast-growing birds produce more feces which make the litter dirtier and causes breast blisters (De Jong et al., 2014). In the present study, it clearly seems that the presence of a plastic floor improved plumage hygiene since the broilers had less contact with feces. Akpobome and Fanguy (1992) and Fraley et al. (2013) observed better (lower) results of feather dirtiness of the broilers for those reared on plastic floors than those reared on wood shavings. On the other hand, Li et al. (2017) reported that an increase in breast blister incidence was observed in birds raised with the perforated flooring system during the summer.

Poor walking ability in birds in broiler meat production is still prevalent, though highly variable between flocks, and indicates potential pain and behavioural restriction. Causes of poor walking ability have multiple factors, but primary risk factors are the high growth rate and poor environmental conditions in broiler houses (Baracho et al., 2012; Bessei, 2006). In this study, there was no incidence of gait scores of 3, 4, or 5, which was associated with poor locomotor quality, in slow-growing broilers (Table 4). Broilers displaying gait scores 1 and 2 had an abnormality in their gait; however, their ability to walk was not severely compromised, and it was considered a moderate condition. However, scores 3 or above are conditions that must be considered important welfare issues, since the locomotor activity of the animals is badly affected (De Jong et al., 2014, 2016; Knowles et al., 2008). In the present study, the similar prevalence of gait scores of 3 or above was found in broilers raised on slatted floors and deep-litter floors (3.6 % of the birds), indicating that animals in this study tended to have less locomotor problems. While there were no lame birds in slow-growing groups (gait score 3 and greater), we found gaits scores 3 or higher in 7.2 % of fast-growing birds in this study. Similarly, Bergmann et al. (2017) reported that Cobb Sasso broilers reared with the alternative husbandry system were more active than conventionally reared Ross 308 broilers.

The avoidance distance test was used to try to determine the human–broiler relationship. This is also referred to as the “touch test” because the idea is to see if you can get close enough to touch the birds. Any changes in the environment and lack of exposure or contact to humans can cause fearfulness in broilers and leads to increased damage and high mortality (Coleman and Hemsworth, 2010; Jones and Boissy, 2011; Li et al., 2017). Bird age also affects the outcome of the avoidance distance test; birds' activities and desire to move significantly drops after 5 weeks of age with increased weight (Bokkers and Koene, 2003). Li et al. (2017) showed that avoidance distance test scores of the bird in two different houses were similar and indicated that the fearfulness and stress levels in both houses were similar. Hemsworth et al. (1994) found that there was a significant relationship between the behavioural responses of birds to an experimenter and feed conversion and suggest that fear of humans may be an important factor limiting the productivity of commercial broiler chickens. In this study, slower growing broilers had a longer distance for the avoidance distance test (22.16 cm) compared with the fast-growing broilers (3.4 cm).

The duration of tonic immobility indicates fear levels of the birds, and some feed additives can prolong duration of tonic immobility in broilers (Ghareeb et al., 2014).

In this study, tonic immobility reaction times were longer in slow-growing broilers (122.2 s) than fast-growing broilers (36.3 s). We assume that this was because slower growing broilers were more active that fast-growing broilers. However, more behavioural traits should be examined, e.g. dust bathing, standing, and walking behaviours to make a better comparison. We could not do this since we did not have enough capacities in our experimental unit. Floor type did not affect both behavioural parameters (avoidance distance test and tonic immobility).

## 5 Conclusions

As a conclusion, it can be said that slow-growing broilers had better welfare parameters and slat flooring could be beneficial to improve animal welfare in broiler production. However, further investigations are needed to evaluate the behavioural needs like dust bathing as well as other active and locomotory behaviours of the broilers in slatted floors.

## Data Availability

The data of this study can be accessed from the corresponding author upon a reasonable request.
